# Offspring sex preferences among patrilineal and matrilineal Mosuo in Southwest China revealed by differences in parity progression

**DOI:** 10.1098/rsos.160526

**Published:** 2016-09-14

**Authors:** Siobhán M. Mattison, Bret Beheim, Bridget Chak, Peter Buston

**Affiliations:** 1 Department of Anthropology, University of New Mexico, MSC 01, Albuquerque, NM 87111, USA; 2 Max Planck Institute for Evolutionary Demography, Leipzig 04103, Germany; 3 Center for Human Genetic Research, Massachusetts General Hospital, 55 Fruit Street, Boston, MA 02114, USA; 4 Department of Biology, Boston University, 5 Cummington Mall, Boston, MA 02215, USA

**Keywords:** kinship, parity progression, fertility behaviour, microdemography, human behavioural ecology

## Abstract

Son preference predominates in China, yet there are patterned exceptions to this rule. In this paper, we test whether lineality (patrilineal versus matrilineal inheritance and descent) is associated with son versus daughter preference among the ethnic Mosuo (Na) of Southwest China. Our results show (i) an increased probability of continued fertility among matrilineal women after having a son compared with a daughter and (ii) an increased probability of continued fertility among patrilineal women after having a daughter compared with a son. These results are consistent with son preference among patrilineal Mosuo and more muted daughter preference among the matrilineal Mosuo. Furthermore, we show (iii) the lowest probability of continued fertility at parity 2 once women have one daughter and one son across both systems, suggesting that preferences for at least one of each sex exist alongside preferences for the lineal sex. The Mosuo are the only known small-scale society in which two kinship systems distinguish sub-groups with many otherwise shared cultural characteristics. We discuss why this, in conjunction with differences in subsistence, may shed light on the evolutionary underpinnings of offspring sex preferences.

## 1. Introduction

Son preference predominates in East Asia (e.g. [1]) and is especially strong in China, as evidenced by excess female childhood mortality, sex-selective abortion and high sex ratios [1–3]. Indeed, despite efforts to mitigate son preference, China's sex ratio at birth (SRB) remains the highest in the world at approximately 120 males born for every 100 females [4]. This is a concern to demographers and policy-makers of Asia, not least because son preference is thought to result in downstream effects whereby violence among men (e.g. [5,6]) and against women (e.g. [7]) increases (but see [8]). Identifying correlates of son and daughter preference is of interest in attempts to mitigate against unwanted effects.

The predominance of son preference belies its uneven and patterned display (e.g. [9]): rural areas often show stronger evidence of son preference (e.g. [10,11]) while areas with increased female economic independence have shown attenuated son preference [12]. One factor associated with variation in son preference is kinship system [1,13]. The patrilineal joint family systems that typified many parts of East Asia have been associated with strong son preference (e.g. [1,14,15]), whereas daughters are often preferred within more sex egalitarian family systems (e.g. [16,17]).

In this paper, we build on these findings to investigate whether kinship system (patrilineal versus matrilineal descent and inheritance) is associated with son or daughter preference or mixed preferences (see below) among the Mosuo (aka Na) of Southwest China. As the world's only small-scale society practising distinct modes of kinship in adjacent communities despite many otherwise shared cultural phenotypes, the Mosuo test enables us to investigate fine-scale differences in offspring sex preferences while controlling for some effects of culture often assumed to be the major determinant of sex preference in Asia and elsewhere. Indeed, even though the association between kinship system and offspring sex preferences is reported both qualitatively (e.g. [1]) or at larger scales of analysis (e.g. regionally [16]), systematic evidence at the individual level is currently lacking. Furthermore, cultural transmission arguments have posited unidirectional diffusion of son preference across China [18], such that the relationship between kinship and offspring sex preference is not a foregone conclusion. This paper's aim is to describe the pattern of offspring sex preferences as a first step on the road to testing evolutionary hypotheses, both cultural and material, of underlying causes.

We use parity progression ratios (PPRs) to explore the relationship between kinship system and offspring sex preferences. The PPR is simply the likelihood of continued fertility at a given parity given that one has arrived at that parity [19]. It has been used in several studies to measure offspring sex preferences [20–22] and has been cited as demographic evidence of overwhelming son preference in China (e.g. Guo 2000, in [23]). Whereas deviations from sex ratio are difficult to establish in small populations [24], PPR may be more suitable. For example, continuing fertility until a desired number of offspring of one sex has been achieved can be evident in PPR without aggregate differences in sex ratio. Moreover, gender preferences may be displayed unevenly across parities and PPRs can illustrate whether preferences are apparent at all parities or whether more complex patterns of preferences are implied (e.g. [14]).

**Figure 1.**
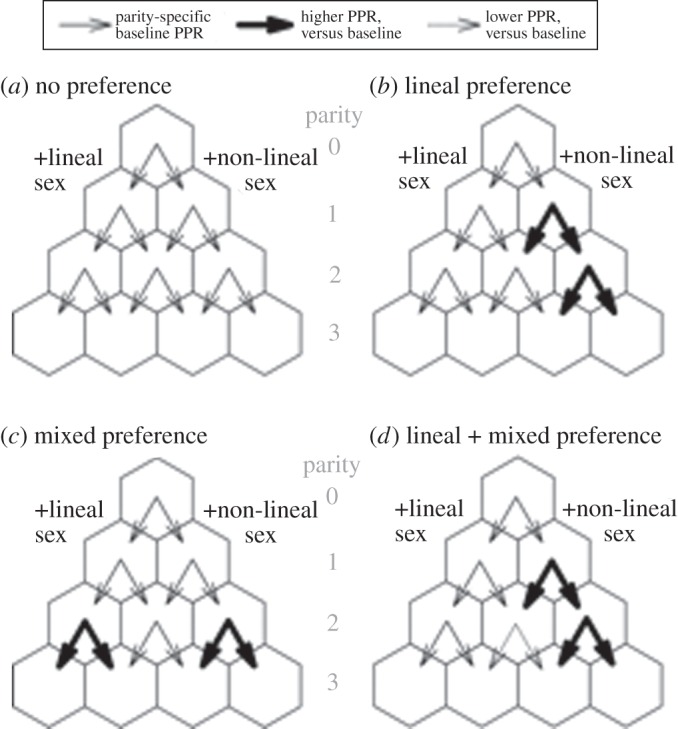
Visual representation of alternative progression scenarios under different types of sex preference. Probability of parity progression for four different preference models in a kinship system. Here we do not specify any baseline probability of reproduction but instead emphasize the probability of progression relative to others at the same parity level. (*a*) H0 – Under the null model of ‘no preference’, continued fertility is equally likely following all parity-sex compositions. (*b*) H1 – Under a ‘lineal preference’ model, women at parities 1 and 2 are more likely to continue to a higher parity if they do not have a child of the lineal sex, compared to other women at the same parity. (*c*) H2 – In a ‘mixed preference’ model, women prefer at least one child of each sex and are more likely to continue reproducing at parity 2 if they have one child of each sex, regardless of lineality. (*d*) H3 – In a ‘lineal + mixed’ preference model, women at parity 1 are more likely to continue reproducing if their child is of the non-lineal sex. At parity 2, both a preference for lineal sex children and a preference for a mixture of sexes is exhibited, such that women with two children of the non-lineal sex are most likely to reproduce again, followed by women with two of the lineal sex. Women with a son and a daughter are thus least likely to continue to reproduce at parity 2. The thickness of arrows and corresponding shading represent the likelihood of progression to a subsequent cell relative to other individuals at the same parity and contingent on having arrived in that cell. We make no assumptions about the optimal number of children.

To determine whether fertility behaviour is associated with the sex composition of the existing set of children and whether this association differs across matrilineal and patrilineal kinship systems, we test among the following plausible hypotheses (figure 1): (H0) No difference—the null hypothesis is one of no preference implied by parity progression. Here, progression rates should be the same regardless of the sex composition of existing offspring. (H1) There is a bias for at least one of the lineal sex—this pattern anticipates higher parity progression when the sex composition of existing children does not include a member of the lineal sex; (H2) there is a preference for at least one of each sex—this pattern anticipates the lowest parity progression after a balance of sexes has been achieved versus no difference in progression following any alternative offspring sex composition. (H3) There is a combined preference in which parents prefer at least one of the lineal sex alongside a preference for one of each sex—this pattern anticipates low parity progression following the birth of a child of the lineal sex *and* low progression following achieving a balance of sexes.

## 2. Material and methods

### 2.1. Permission to carry out fieldwork

Research protocols were reviewed and approved by the University of Washington Institutional Review Board (UW IRB 07-4858-C 01) and as part of obtaining research permission locally in China (via the Yunnan Academy of Social Sciences). In addition, we visited local police stations to describe the purposes of our research and discussed all methods with local town heads prior to the onset of fieldwork. Voluntary informed consent was obtained from all participants in the study and the fieldwork team has been continuously available to participants for subsequent contact.

### 2.2. Population

Although the Mosuo are best known for their matrilineal kinship system [13,25–30], they in fact consist of two distinct subpopulations differing in geography, kinship and subsistence [31,32]. Mosuo residing in the basins of the Hengduan Mountains practise matrilineal descent and inheritance, preferentially engaging in a non-marital reproductive union known as *sese* (literally ‘walking back and forth’; Mandarin 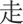

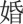
: *zouhun*––‘walking marriage’) [28,33], and in natalocal post-marital residence, in which reproductive partners remain in their natal matrilineal households [25,28]. Patrilineal Mosuo reside in distinct geographical regions, typically ensconced in smaller houses nestled in steeper regions of the mountains (electronic supplementary material, figure S2). Although they share much in common with their matrilineal counterparts, including language, attire, certain customs, religious beliefs and even blood relations, they differ almost entirely in their systems of inheritance and descent, and in marriage norms [28,31,32]. Lineage membership and transmission of resources occur via the male line rather than female among the patrilineal Mosuo; marriage is normative and post-marital residence is typically patrilocal. Subsistence also differs: although both subpopulations practise subsistence horticulture or low intensity agriculture and both keep a variety of livestock and fowl, the patrilineal Mosuo reside among more circumscribed terrain, and rely more extensively on goats and sheep. These particular differences in kinship and subsistence amidst broader shared cultural characteristics enable a unique comparison in which kinship and subsistence systems can be examined as correlates of son or daughter preference while holding many other cultural factors constant.

Many contemporary Mosuo regulate their fertility using barrier contraceptives and ultimately tubal ligation to prevent further pregnancies. They are subject to fertility restrictions of the Chinese fertility policy: as a minority population, they are allowed to have a maximum of three children. Implementation of the fertility policy has been highly variable across China [9], but has resulted in a dramatic decline in Mosuo fertility as per elsewhere in China (e.g. [13]). It is possible that this has intensified the expression of sex preference [18], including through distortions of the secondary sex ratio (i.e. through sex-selective abortion or infanticide) [9]. Exceptionally large datasets are required to analyse secondary sex ratios [24]; we focus instead on the more tractable question of how fertility behaviour is patterned according to the sex ratio of existing offspring sets.

### 2.3. Data

Data in this study are drawn from a demographic survey of 228 households conducted in 2008 by S.M.M. The survey covered 12 villages, seven matrilineal and five patrilineal, at varying distances from Yongning, the township seat and largest local market. One or more adults in each household responded to each survey. All households within walking distance were surveyed, resulting in a partial or complete census of surveyed villages. All surveys were administered in Mandarin Chinese or the local dialect by a member of the research team, or translated into Naru, the Mosuo language, by a local assistant.

Each respondent provided information on all individuals who were considered to be members of the household—in this case, all individuals who were born in the household (even if residing elsewhere at the time of the survey). Surveys solicited socio-demographic information on household members, including monthly income (individual and household), source of income, asset holdings (e.g. land, durable goods) approximate age, sex, occupation, education and so on [34]. Only adult women currently residing in the study area are considered in these analyses.

### 2.4. Variables and analysis

See the electronic supplementary material for additional description of methods and descriptive statistics. Initial explorations of the association between kinship systems and offspring sex preference were examined using PPRs. Specifically, we calculated the probability of progressing to a subsequent birth at the first and second parities, broken down by the sex composition of the existing offspring at each parity. Subsequent parities' sample sizes were small and are not considered in our analyses except as required for the simulation (see below). Furthermore, the Mosuo, as a minority population, are allowed up to three children under the Chinese fertility policy; thus we expected more continuity between pre- and post-policy implementation cohorts at early parities (but we include a control for cohort in our inferential analyses; see below).

### 2.5. Inferential analysis

Following evidence that the influences on fertility behaviour vary strongly by parity (e.g. [14,35]), we constructed two models, one for the progression from parity 1 to 2 and a second from parity 2 to 3. To estimate the association between fertility and parity progression in each kinship system, we modelled each woman's annual birth ‘outcome’ (‘1’ if child born, ‘0’ if not) by a logistic regression on her age, birth cohort, predominant lineality in village (i.e. patrilineal versus matrilineal), current offspring sex composition, whether the woman earns wages in a ‘market-integrated’ job and years of formal education. These variables (see also the electronic supplementary material) are included because they are suspected or known to have effects on fertility behaviour in this population. Specifically, fertility has decreased over time [13], in part due to the effects of the Chinese fertility policy. Age affects fertility via changes in fecundability and reproductive behaviour [36,37]. Increased socio-economic status, including employment and education, is typically associated with decreased fertility in societies undergoing fertility transition, including the Mosuo (e.g. [38]). We fit separate models that included these covariates as main effects, several interaction terms (table 1), as well as a random intercept for mothers to control for dependency in the data; these models were run separately for women at parity 1 (who either have a son or a daughter) and parity 2 (who have either at least one child of the lineal sex or no children of the lineal sex). Birth cohorts were defined by decade from 1920 to 1980, while age was subdivided into quinquennials. Baseline cohort and age groups were chosen by plurality of observations, and the 1980–1989 cohort was excluded from parity 2 models due to small sample size, with no change in substantive results. There were 1860 trials for *n* = 385 women for parity 1, and 2318 trials for *n* = 282 women at parity 2. All models were coded in R and fit in STAN via the map2stan function in the *rethinking* package [39].

**Table 1.** Logistic regressions of annual probability of birth.

	parity 1 (M1)		parity 2a (M2)		parity 2b (M3)	
predictor	B	95% HPDI	B	95% HPDI	B	95% HPDI
intercept	−0.54	(−0.99, −0.05)	−2.38	(−2.94, −1.81)	−1.90	(−2.53, −1.27)
son^a^	0.42	(0.01, 0.81)				
no lineal kids^b^			0.50	(−0.09, 1.17)		
balanced sex ratio^c^					−0.71	(−1.24, −0.16)
patrilineal area^d^	1.17	(0.50, 1.84)	−0.74	(−1.54, 0.00)	0.14	(−0.72, 0.97)
age 15–19	−2.03	(−2.94, −0.98)	−3.38	(−6.14, −0.78)	−3.19	(−6.02, −0.71)
age 20–24	(baseline)		0.35	(−0.26, 1.01)	0.49	(−0.19, 1.12)
age 25–29	−0.11	(−0.46, 0.30)	(baseline)		(baseline)	
age 30–34	−0.79	(−1.36, −0.23)	−0.98	(−1.51, −0.42)	−1.02	(−1.54, −0.45)
age 35–39	−1.00	(−1.74, −0.22)	−2.93	(−3.95, −1.95)	−3.02	(−4.06, −1.99)
age 40–46	−2.38	(−4.31, −0.76)	−5.25	(−7.65, −3.05)	−5.32	(−7.97, −3.08)
cohort 1920–1929	−1.44	(−2.45, −0.35)	2.46	(1.03, 3.73)	2.52	(1.11, 3.75)
cohort 1930–1939	−0.81	(−1.55, −0.02)	1.39	(0.46, 2.28)	1.49	(0.59, 2.23)
cohort 1940–1949	−0.29	(−0.89, 0.33)	2.25	(1.55, 3.07)	2.21	(1.46, 2.85)
cohort 1950–1959	0.14	(−0.46, 0.76)	1.55	(0.90, 2.30)	1.52	(0.82, 2.29)
cohort 1960–1969	(baseline)		(baseline)		(baseline)	
cohort 1970–1979	−0.99	(−1.58, −0.53)	−2.79	(−3.99, −1.65)	−2.64	(−3.91, −1.59)
cohort 1980–1989	−2.62	(−3.62, −1.71)				
market-integrated job	−1.11	(−2.86, 0.54)	1.25	(−1.97, 4.32)	1.52	(−1.69, 4.73)
highest grade level	−0.04	(−0.12, 0.03)	−0.04	(−0.19, 0.11)	−0.04	(−0.19, 0.10)
birth last year	−1.99	(−2.55, −1.48)	−2.58	(−3.51, −1.77)	−2.53	(−3.46, −1.69)
Son × Patrilineal area	−1.17	(−2.10, −0.29)				
No lineal kids × Pat. area			1.79	(0.32, 3.15)		
Balanced SR × Pat. area					−0.79	(−2.02, 0.31)
mother random effect	1.00	(0.70, 1.35)	0.86	(0.34, 1.30)	0.78	(0.39, 1.34)

^a^Baseline category is women with one daughter.

^b^Baseline category is women with at least one child of lineal sex, by kinship system.

^c^Baseline category is women with two boys or two girls.

^d^Baseline category is women in matrilineal.

To assess the progression behaviour implied by the logistic regression models of fertility, we fit additional logistic regression models for nulliparous women and women at parities 3 and 4, and then generated 1000 simulated populations (see the electronic supplementary material for additional details) that sample from these parity models to determine their fertility schedules. These simulations allowed us to estimate the probability that a woman continues to reproduce at a particular parity level and sex composition, conditional on her having reached it.

## 3. Results

**Figure 2.**
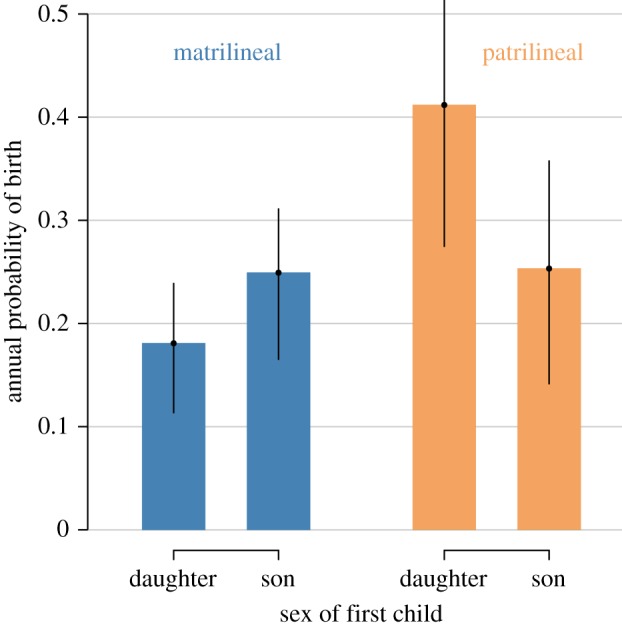
Annual probability of birth, by kinship system and sex composition at parity 1. Values apply to women born between 1970 and 1979 when they are aged 20–24. Additional controls include market-integrated employment and years of education (table 1). Error bars show the 89% HPDI for each parameter estimate.

At parity 1, among matrilineal villages, we estimate that the annual odds a woman with one son will have another birth are 1.5 times the odds a woman with one daughter will have another birth (OR = exp(0.42), table 1/M1 and figure 2). This relationship reverses for women in patrilineal villages, in which the odds of another birth by a woman with one daughter is 2.1 times the odds of another birth by a woman with one son (OR = 1/exp(0.42 − 1.17)). Overall, evidence at parity 1 in both communities rejects H0, supports H1 or H3, but says nothing about H2 (figure 1). The preference would seem to be more muted for matrilineal Mosuo compared to patrilineal.

**Figure 3.**
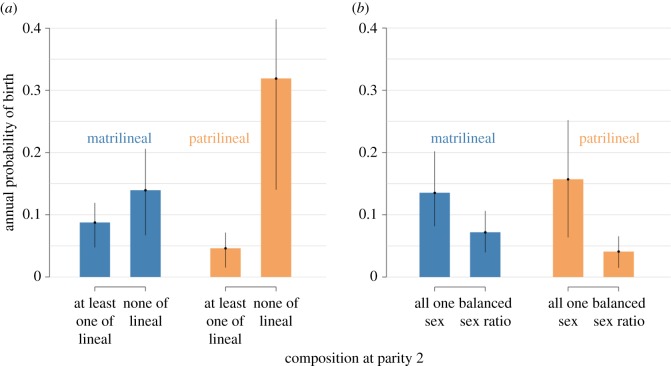
Annual probability of birth, by kinship system and sex composition at parity 2. Offspring sex composition is coded in two alternative ways, corresponding to models 2 and 3 in table 1. Outcome probabilities apply to women born between 1960 and 1969 when they are aged 25–29. Additional controls include market-integrated employment and years of education (table 1). Error bars show the 89% HPDI for each parameter estimate.

At parity 2, the odds of continued reproduction were also higher among women without children of the lineal sex in both matrilineal and patrilineal communities (M2, table 1/M2 and figure 3*a*). Specifically, in matrilineal women, we estimate the odds of continued reproduction for women with no daughters are 1.6 times higher than the odds of continued reproduction for women with at least one daughter. Similarly, in patrilineal women, the odds of continued reproduction are more than nine times higher for women with no sons compared with women with at least one son. Again, this rejects H0, provides support for H1 or H3, but does not speak to H2.

Analysing the parity 2 data in a slightly different way, in both matrilineal and patrilineal communities, the lowest odds of continued fertility were seen among women who had one child of each sex (M3, table 1/M3 and figure 3*b*). Specifically, among matrilineal women, the odds of continued fertility for women with two children of the same sex was twice as large as the odds of continued fertility for women with one son and one daughter (1/exp(−0.71)); among patrilineal women, the odds of continued fertility for women with two children of the same sex was four times as large as the odds of continued fertility for women with one son and one daughter (1/exp(−0.71 − 0.79)). Bayesian 89% highest posterior density intervals (HPDIs) assign almost no density past zero, indicating high confidence in the direction of these differences. Probabilistic interpretations are presented in figure 3*b*. This result falsifies H0 once more and provides support for H2 at parity 2, but not parity 1, and overall, for H3.

**Figure 4.**
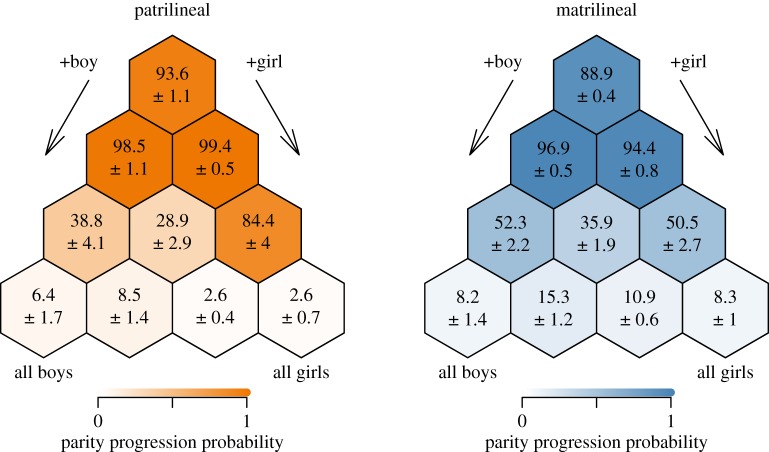
Simulated PPRs by parity level and sex composition. The expected percentage of women that will continue reproducing in a given parity-sex configuration (hexagonal cell), out of those who will reach it, is given with the empirical standard deviation across simulations.

PPRs apparent in simulations derived from these models are displayed in figure 4. As suggested by the models, patrilineal women are significantly more likely to continue reproduction at parity 1 if they have had a daughter (a 99.4% chance) as opposed to a son (a 98.5% chance). In matrilineal communities, however, women are more likely to continue after one son, 96.9% versus 94.4% after one daughter. Thus, although most women are inclined to have a second birth, there is a significant difference in both communities in continuation based on the sex of the prior offspring. This bias is continued at parity 2 for the patrilineal simulations, with daughter–daughter women progressing much more often than son–son women; this bias is less apparent in matrilineal simulations with son–son women continuing about as often as daughter–daughter women. Most remarkably, women are least likely to continue reproduction at parity 2 in both matrilineal and patrilineal groups if they have one son and one daughter. Overall, figures 2–4 reveal parity progression patterns that are consistent with a scenario that reflects combined preferences for a balance of sexes (or for at least one of each sex) and for a preference of at least one child of the lineal sex (H3).

## 4. Discussion

Our results provide evidence of differences in offspring sex preferences across the first two parities among Mosuo women in Southwest China, including marked differences by kinship system (figure 2). Continued childbearing after parity 1 was associated with the sex composition of existing children, as anticipated: women were more likely to continue bearing children after having a son in matrilineal areas and after having a daughter in patrilineal areas (supporting H1, figure 1). Parity 2 similarly showed evidence consistent with this hypothesis: women without children of the lineal sex were much more likely to continue reproduction compared with women who had had at least one child of the lineal sex (supporting H1, figure 3*a*). Finally, in both matrilineal and patrilineal areas, mothers were the least likely to continue bearing children after having a balance of sexes (supporting H2, figures 3*b* and 4). The total evidence suggests support for both H1 and H2, implying support for H3, a preference for a mix of sexes (or at least one of each sex), combined with a preference for at least one of the lineal sex (figures 1 and 4).

That kinship system and subsistence can be isolated among other shared characteristics of these subpopulations suggests several plausible interpretations. First, it is possible that kinship system, as part of the local cultural ecology, produces son or daughter preference. In this view, kinship represents a domain of culture that causes son or daughter preference. This cause can be inferred wherever authors conclude that altering norms (e.g. via education or public messaging) diminish son or daughter preference within a given sociological system [1,40]. Note that this explanation is potentially inconsistent with regional cultural transmission models that see preferences for offspring of a given sex as transmitted via cultural diffusion [18] to the extent that such models ignore the role of kinship in mediating the pattern of diffusion. While both arguments are consistent with cultural evolutionary models of behaviour change, the former would predict an association between kinship system and offspring sex preference, whereas the latter would predict a more universal diffusion of son preference across China in the absence of preventative factors.

A second possibility is that the differences in subsistence and geography (i.e. the local environmental ecology) explain the observed relationship. Such a view is consistent with predictions resulting from evolutionary hypotheses that envision offspring sex preferences as strategies that allow parents to maximize reproductive benefits accruing from the investments in their children, which vary with ecological context [41–43]. Several evolutionary models offer explanations for why parents might prefer a given sex (e.g. [41,42,44]) and we do not attempt to adjudicate among them here (but see, e.g. [45,46] for empirical tests of alternatives). We suspect, however, that a generalized Trivers–Willard effect [13,42,47,48] may lie at the root of the differences revealed by the data. This is supported by previous results [13], which showed an association between wealth in land and reproductive success in the matrilineal Mosuo that was consistent with higher marginal returns to parental fitness associated with investing land in daughters. Whether this effect is upheld among the patrilineal Mosuo remains to be tested, but evidence is mounting that supports this hypothesis as an underlying cause of inheritance patterns in other cultures, albeit at lower resolution [13,44,47,49–51].

A third possibility is that cultural and ecological evolutionary explanations complement each other, with the cultural norm acting synergistically with the strategy that produces the highest fitness returns. This seems the most plausible and satisfying explanation of the Mosuo case. The matrilineal Mosuo communities have long existed against a backdrop of son preference in neighbouring communities, which consist mainly of patrilineal Han, Pumi, Yi and Naxi [40] yet have not shown evidence of son preference. This suggests that, although historically there have been opportunities for cultural transmission, the matrilineal Mosuo were previously unreceptive to other cultural models. At the same time, demographic evidence suggests significant departures from matrilineal norms in areas where tourism generates the most income [27], suggesting that changes in the means of production have increased receptivity to novel cultural norms (i.e. increased valuation of sons). The idea that evolutionary ecological and cultural evolutionary models are synergistic in this case resonates with other studies showing that changes in economic returns to daughters coincide with shifts in their valuation [40] and recognizes that kinship is not a monolithic feature of a local culture. Future studies would do well to incorporate cultural and evolutionary ecological models of behavioural change, which could perhaps be explored using the actual versus perceived value of different offspring in different contexts.

In both matrilineal and patrilineal kinship ecologies, preferences for the lineal sex were shown to exist alongside preferences for at least one of each sex, as mothers were most likely to cease childbearing after giving birth to one son and one daughter (figure 3*b*). Indeed, previous qualitative evidence [31] revealed stated preferences for a balance of sexes in both areas. To the best of our knowledge, despite a general expectation in evolutionary studies of equal investments in the sexes [52], there are no systematic evolutionary studies showing evidence that supports preferences for both sexes alongside a preference for a single sex. For example, rejections of the Trivers–Willard Hypothesis (e.g. [53,54]) support equal investment in the sexes in populations where wealth is not differentially associated with reproductive success in sons versus daughters. Other tests of preferences for one sex (e.g. [45,55,56]) have focused on the relative costs or benefits of that sex as affected by specific socio-ecological circumstances. We urge future evolutionary studies that explore how preferences are patterned across socio-ecological contexts and that consider complex sets of preferences and the distribution of preferences across parities [14,41].

This study suggests that kinship is an important, but not uniform, predictor of sex preferences. At both parities, the magnitude of the differences in parity progression was higher in the patrilineal areas compared with the matrilineal ones. We speculate that this is due to the potential differences in reproductive, social and economic returns earned through sons versus through daughters (e.g. [57,58]). Variance in reproductive success and relative reproductive potential is higher in males in the vast majority of societies [59], suggesting that, much of the time, investments in men produce steeper returns than similar investments in daughters [44,50,51]. Thus, it may be reasonable to expect relatively muted preferences in cases of matriliny if variance in reproductive success among males in patrilineal societies is higher than in matrilineal societies. We suspect that the steeper terrain and stronger focus on goats and sheep as the main forms of livestock among patrilineal Mosuo may result in stronger gains to male-biased inheritance and higher variance in male reproductive success [34,44,57,60,61]. It is also possible that matrilineal preferences are relatively muted, because they run in opposition to prevailing norms for son preference. This is the subject of ongoing investigation and data collection and remains to be tested. Regardless, our results are consistent with at least one other comparative study of parity progression of two different matrilineal and patrilineal populations (R Sear 1997, unpublished analyses) and more broadly with the finding that matrilineal societies tend to be more egalitarian than patrilineal ones [62], pointing to the need for more theoretical and empirical efforts in this area. Our result underscores a key premise of behavioural ecology that behaviours are expected to be sensitive to local ecologies (see also [51]) and that cultural norms and institutions are shaped by socio-ecological circumstances [63].

## 5. Conclusion

We have argued in this paper that human reproductive decision-making can be a nuanced response to local socio-ecology, as predicted by various streams of evolutionary theory. Although most evolutionary models of offspring sex preference have focused on the adaptive value of sons versus daughters (reviewed in [41]), it is undoubtedly the case that both sexes provide reproductive and economic benefits to their parents, in context-specific ways. This should lessen the effect of sex preference on fertility decisions, but is unlikely to eliminate it where socio-economic or cultural factors lead to differences in the perceived or real utility of reproduction of sons versus daughters.

PPRs are commonly used in demographic and sociological research to explore the effects of the sex composition of children on parental fertility (e.g. [64]); they are increasingly incorporated into anthropological demography [14,22,65], but are far less common than sex ratios or more direct proxies of parental investment in a given sex in evolutionary anthropological research. In this paper, we have shown that the rates of parity progression differ according to kinship system (patrilineal versus matrilineal) among the Mosuo of Southwest China. These results challenge claims that son preference is ubiquitous in East Asia (see also [66]) and provide additional support for evolutionary anthropological models that link kinship and sex-biased parental investment. Assimilation into a larger mainstream culture (e.g. via education) if not sufficient to explain the patterns of son preference in Asia, may interact in important ways with social, material and fitness incentives contributing to offspring sex preferences. Models that incorporate these effects would shed light on policies designed to mitigate undesirable consequences of unequal inheritance [49].

## Supplementary Material

Supplementary Information

## Data accessibility

Our data (doi:10.13140/RG.2.1.2440.4724) are deposited on S.M.M.'s Researchgate page, accessible via the following link: http://bit.ly/2bcjDj9.
