# Supplementary material for: Offspring sex preferences among patrilineal and matrilineal Mosuo in Southwest China revealed by differences in parity progression
Source: R Soc Open Sci. 2016 Sep 14;3(9):160526. doi: 10.1098/rsos.160526 (PMC5043333; doi:10.1098/rsos.160526)
Supplement: Supplementary Information [file rsos160526supp1.docx]

**Supplementary Information**

**Population**

The Mosuo (aka Moso, Na) are a population of approximately 40,000 minority Chinese agriculturalists [1] situated near Lugu Lake on the border of Yunnan and Sichuan Provinces where they have lived continuously since the mid-13^th^ century [2] (Figure S1). They exist as two distinct subpopulations (matrilineal and patrilineal) adhering to different kinship systems and living in distinct geographic regions in the Hengduan Mountains in Yunnan and Sichuan provinces [3,4]. Among matrilineal Mosuo, inheritance is transmitted via the female line, to all descendants of any females residing in the household. Thus, while men have usufruct access to their mothers’ and sisters’ resources during their lifetimes, because men’s children are part of their partners’ lineages, long-run resource transmission is solely matrilineal [5]. The matrilineal Mosuo are best known for a system of pairing known as *sese,* (Mandarin: *zouhun* –– ‘walking marriage’), which, in its traditional mode, involves a man visiting a woman at night for sexual relations and secretly absconding to his natal household in the early morning [6]. The only prerequisite for a *sese* relationship is mutual consent between the man and the woman, and a *sese* relationship can be terminated at any time for any reason. The role of the biological father is normatively reduced and maternal uncles in matrilineal Mosuo families are said to bear primary responsibility for children. Though there is evidence of contemporary paternal investment in children [7], this type of non-marital union may be associated with lower paternity certainty than is likely under systems of institutionalized marriage [8]. A Mosuo woman has no obligation to commit to a single sexual relationship nor can she be compelled to provide evidence of paternity. Relatively low paternity certainty, in conjunction with a resource base that does not result in higher returns to male RS, has been argued to contribute to daughter-biased investment and

| 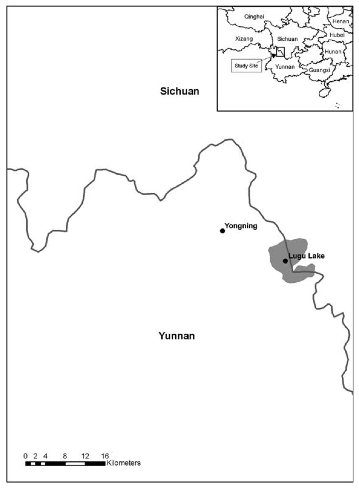 |
| --- |
| **Figure S1. Map of the study region.** The Mosuo reside on the border of Sichuan and Yunnan Provinces in Southwest China. Lugu Lake is the main locus of tourism, with more agricultural households residing near Yongning. |

matrilineal kinship among the Mosuo [5]. If this is correct, then matrilineal kinship has resulted from socio-ecological conditions that did not tend to, on the whole, favor males. In turn, the kinship system augmented norms that have reinforced the value of daughters. The birth of at least one daughter in each generation is considered necessary for lineage continuity [9], providing an additional rationale for our predictions that matriliny should be associated with evidence of daughter preference.

| **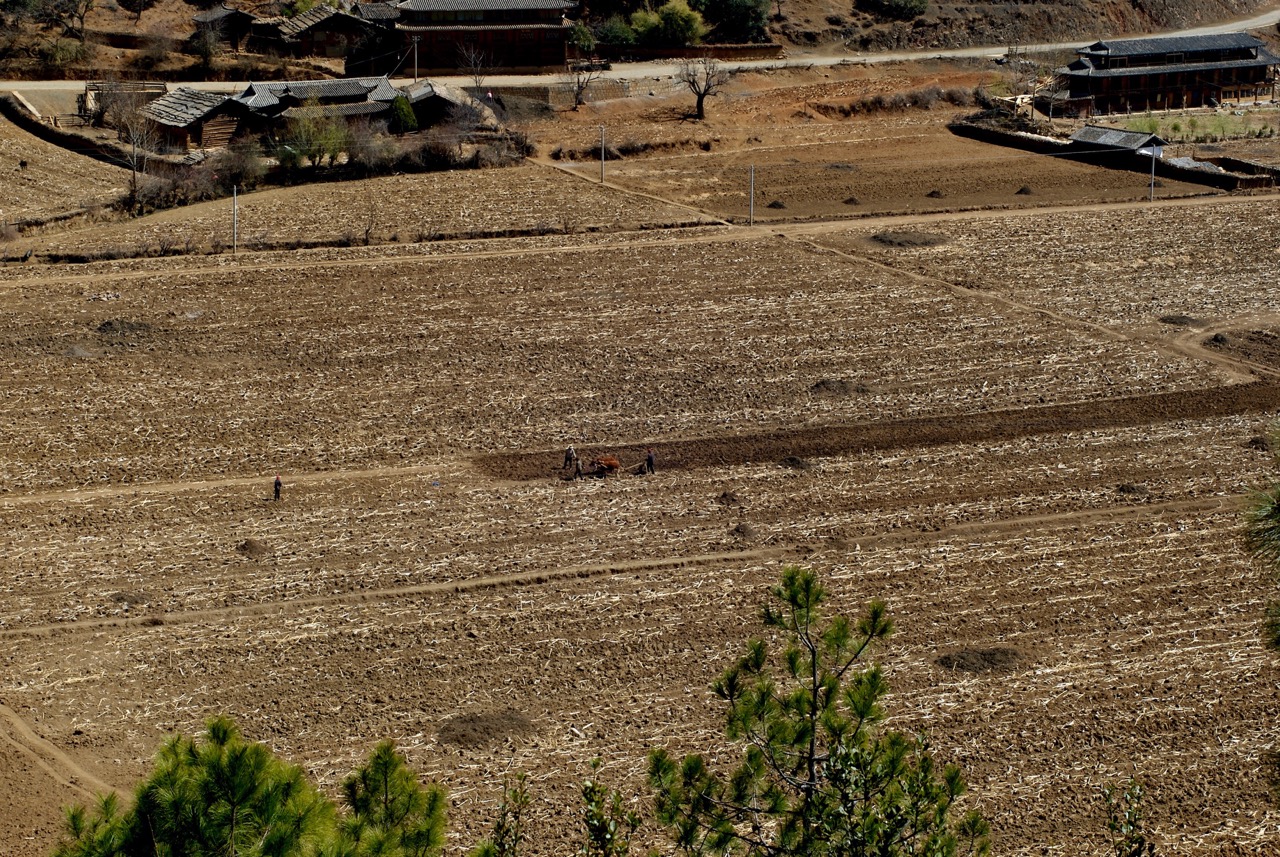** | **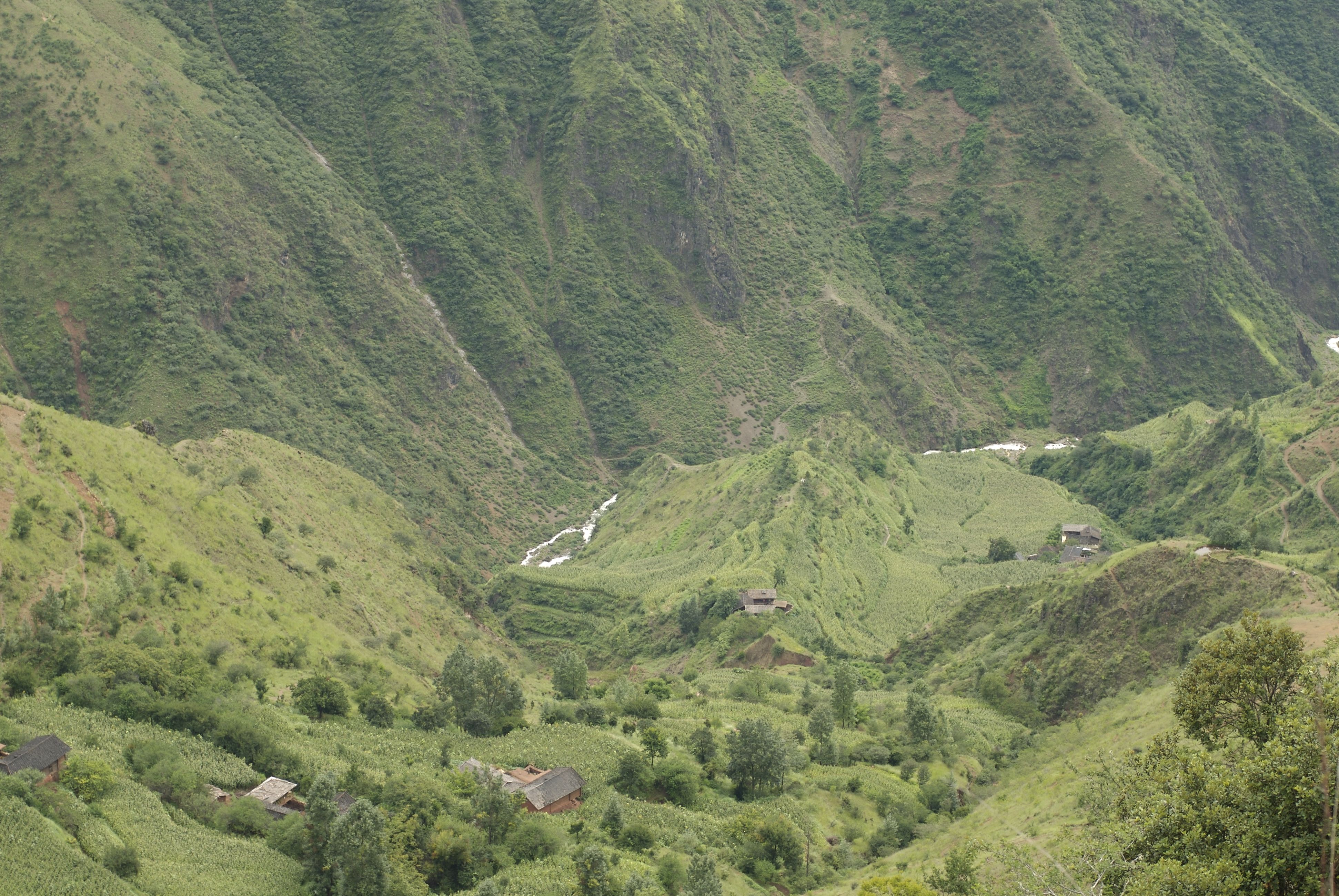** |
| --- | --- |
| **Figure S2. Geography of matrilineal and patrilineal areas.** The matrilineal Mosuo reside in basins of the Hengduan Mountains (left); patrilineal households (right) are scattered within steeper terrain at higher elevations only kilometers away, but a difficult day-long trek by horseback, from the matrilineal areas. | |

Very little is known, ethnographically or demographically, about the patrilineal Mosuo. Fieldwork in 2006 and 2008 by the senior author revealed patterns that were dramatically different from the matrilineal Mosuo, however. In addition to residing in isolated households in steep terrain (Figure S2), the patrilineal Mosuo herd animals such as goats and sheep that are relatively uncommon in the basin areas. Mattison has speculated previously [10] that patrilineality evolved here in conjunction with a resource base that favors males. Briefly, since land is relatively defensible as well as less easily expandable than in the basin areas (i.e. it is relatively economically defensible), and due to the economies of scale possible with pastoral animal herding [11, see also 12], men are likely to receive higher reproductive returns than women [see 13] from land and livestock. Exclusive marriage is normative among the patrilineal Mosuo, which should result in higher paternity certainty, as is primogeniture, wherein the oldest son inherits the family homestead. Thus, as opposed to the matrilineal Mosuo, among the patrilineal Mosuo, lineage continuity depends on the presence of at least one son.

Regardless of their origins, the difference in kinship systems among the Mosuo provides a strong basis for understanding the association between kinship and the expression of gender preferences. Because other aspects of these two subpopulations are shared, including language and many other dimensions of their culture (e.g., similar naming and coming-of-age rituals, shared religion, etc.), the Mosuo case represents a case study in which kinship may be relatively isolated as a potential correlate for differences in sex-biased parental investment.

**Additional Information on Data, Variables, and Analyses**

| **Table S1. Descriptive statistics, by kinship system** | | | | |
| --- | --- | --- | --- | --- |
|  | | Overall | Matrilineal | Patrilineal |
| N Women | | 497 | 405 | 92 |
| Age (years)^a^ | | 41.7 (17.4) | 42.3 (17.5) | 39.0 (16.4) |
|  | |  |  |  |
| Monthly household income (RMB)^a^ | | 2228 (4233) | 2641 (4587) | 403 (332) |
| Number of living children | | 2.1 (1.8) | 2.1 (1.9) | 1.9 (1.3) |
| Proportion of boys among living children | | 47.8% | 46.6% | 52.9% |
| Formal education^b^ | |  |  |  |
|  | None | 300 (60.4%) | 246 (60.1%) | 55 (59.8%) |
|  | Elementary | 84 (16.9%) | 61 (15.0%) | 23 (25.0%) |
|  | Middle | 60 (12.1%) | 50 (12.3%) | 10 (10.9%) |
|  | High | 37 (7.4%) | 34 (8.4%) | 3 (3.3%) |
|  | Post-Secondary | 16 (3.2%) | 15 (3.7%) | 1 (1.1%) |
| ^a^Age is approximated to the nearest lunar year; respondents provided their birth animal and decade of birth  ^b^Formal education is measured as a categorical variable representing progression through the Chinese educational system; see text for details | | | | |

*Variable Operationalization*

**Age**: Each trial outcome in the logistic regression, either resulting in a woman giving birth or not, is associated with the age at which that woman’s trial took place. We use 5-year age categories, each estimating its own intercept, to correspond to known fertility schedules in East Asia that factor into the agent-based simulation (see below). Alternative specifications we attempted included linear, quadratic and cubic age controls. Running models using these specifications did not impact estimations of the kinship x parity composition parameters central to our study, and were excluded on the basis of information-criterion model comparison (<0.001% of AIC weight, compared to final models).

**Birth Cohort**: Several specifications of birth cohort were attempted before settling on decadal categories. We fit quadratic and cubic predictors of age cohort, as well as binary variables for birth before or after 1979 (at which time the fertility policy was implemented locally). Decadal categories were strongly preferred over binary shifts as indicated by AIC, and were chosen over polynomial cohort controls for ease of conceptual understanding and presentation. All main results were preserved across each cohort specification.

**Market-integrated job***:* A woman was defined as having a market-integrated job if she indicated that she was employed in the governmental, educational, or tourism sector. An example of a market-integrated job would be entrepreneurial activities; a non-example would be farming.

**Mother Random Effects**: Because each woman is represented once for each year between age 15 and 45 for which we have data, those women not yet age 45 at time of data collection are under-represented in the sample. This motivated a random-effects approach in which each woman is assigned a unique intercept distributed normally around a population average value according to a random effects variance parameter, which was assigned a Cauchy (0,1) prior.

*Model Specification*

Models were specified in R using map2stan syntax and automatically translated into equivalent STAN specification. All regression coefficients were assigned a vague Gaussian prior with mean 0 and standard deviation 100. Fitting was done by a burn-in of 2,000 iterations followed by a sampling of 2,000 iterations in all three models described in Table 1. Convergence was confirmed by visual inspection of samples and the Gelman-Rubin statistic.

**SI References**

1. Walsh, E. R. 2004 The Na. *Encycl. Sex Gend. Men Women Worlds Cult.*

2. Shih, C.-K. 2010 *Quest for Harmony: The Moso Traditions of Sexual Union & Family Life*. Stanford, CA: Stanford University Press.

3. Shih, C. 1993 The Yongning Moso: sexual union, household organization, gender and ethnicity in a matrilineal duolocal society in Southwest China.

4. Mattison, S. M. 2006 Pilot project: the behavioral ecology of sex-biased parental investment among the ethnic Na of Southwest China.

5. Mattison, S. M. 2011 Evolutionary Contributions to Solving the ‘Matrilineal Puzzle’: A Test of Holden, Sear, and Mace’s Model. *Hum. Nat.* **22**, 64–88. (doi:10.1007/s12110-011-9107-7)

6. Shih, C.-K. 2001 Genesis of Marriage among the Moso and Empire-Building in Late Imperial China. *J. Asian Stud.* **60**, 381–412. (doi:10.2307/2659698)

7. Mattison, Siobhán M., Scelza, Brooke & Blumenfield, Tami 2014 Paternal investment and the positive effects of fathers among the matrilineal Mosuo of Southwest China. *Am. Anthropol.* **116**, 591–610.

8. Mattison, S. M. 2010 Economic Impacts of Tourism and Erosion of the Visiting System Among the Mosuo of Lugu Lake. *Asia Pac. J. Anthropol.* **11**, 159–176. (doi:10.1080/14442211003730736)

9. Shih, C. & Jenike, M. R. 2002 A cultural-historical perspective on the depressed fertility among the matrilineal Moso in Southwest China. *Hum. Ecol.* **30**, 21–47.

10. Mattison, S. M. 2010 Demystifying the Mosuo: The behavioral ecology of kinship and reproduction of China’s ‘last matriarchal society’.

11. Holden, C. J. & Mace, R. 2003 Spread of cattle led to the loss of matrilineal descent in Africa: a coevolutionary analysis. *Philos. Trans. R. Soc. Lond. B. Biol. Sci.* **270**, 2425–2433.

12. Mattison, S. M., Smith, Eric Alden, Mary K. Shenk & Ethan E. Cochrane In press. The evolution of inequality. *Evol. Anthropol. Issues News Rev.* , 2016 (In Review).

13. Trivers, R. L. & Willard, D. E. 1973 Natural selection of parental ability to vary the sex ratio of offspring. *Science* **179**, 90–92.
